# Development of a Multifunction Set Yogurt Using *Rubus suavissimus* S. Lee (Chinese Sweet Tea) Extract

**DOI:** 10.3390/foods9091163

**Published:** 2020-08-24

**Authors:** Mahmoud Abdel-Hamid, Zizhen Huang, Takuya Suzuki, Toshiki Enomoto, Ahmed M. Hamed, Ling Li, Ehab Romeih

**Affiliations:** 1 Guangxi Buffalo Research Institute, Chinese Academy of Agricultural Sciences, Nanning 530001, China; mahmoud.mohamed@agr.cu.edu.eg (M.A.-H.); hzz90302@163.com (Z.H.); 2 Dairy Science Department, Faculty of Agriculture, Cairo University, Giza 12613, Egypt; ahmed.hamed@agr.cu.edu.eg; 3 Department of Biofunctional Science and Technology, Graduate School of Biosphere Science, Hiroshima University, 1-4-4 Kagamiyama, Higashi-Hiroshima 739-8528, Japan; takuya@hiroshima-u.ac.jp; 4 Department of Food Science, Ishikawa Prefectural University, Nonoichi, Ishikawa 921-8836, Japan; enomoto@ishikawa-pu.ac.jp

**Keywords:** *Rubus suavissimus* S. Lee (Chinese sweet tea), yogurt, antioxidant, anticancer, antihypertensive

## Abstract

*Rubus suavissimus* S. Lee leaves, also known as Chinese sweet tea or Tiancha, are used in folk medicine in southern China. This study evaluated the impact of the addition of Chinese sweet tea extract (0.25%, 0.5%, and 1%) on the chemical composition, organoleptic properties, yogurt culture viability, and biological activities (i.e., antioxidant, anticancer, and antihypertensive activities) of yogurt. Seven phenolic compounds were reported in Chinese sweet tea for the first time. The numbers of the yogurt culture were similar across all yogurt treatments. The yogurt supernatant with 0.25%, 0.5%, and 1% Chinese sweet tea extract had a total phenolic content that was 3.6-, 6.1-, and 11.2-fold higher, respectively, than that of the control yogurt. The biological activities were significantly increased by the addition of Chinese sweet tea extract: Yogurt with the addition of 1% Chinese sweet tea extract had the highest biological activities in terms of the antioxidant activity (92.43%), antihypertensive activity (82.03%), and inhibition of the Caco-2 cell line (67.46%). Yogurt with the addition of 0.5% Chinese sweet tea extract received the highest aroma and overall acceptability scores. Overall, Chinese sweet tea extract is a promising food ingredient for producing functional yogurt products that may substantially contribute to reducing the risk of developing chronic diseases such as cancer and cardiovascular disease.

## 1. Introduction

Phytochemicals are produced by plants as secondary metabolites to protect themselves from microbial attack and to control environmental stress. Among these phytochemicals, phenolic compounds exhibit various biological activities, including antioxidant, anticancer, antiviral, anti-allergic, antihypertensive, anti-inflammatory, and antidiabetic activities. These compounds are commonly present in vegetables, cereals, herbs, fruits, and green and black teas; due to their biological activities, they are used as natural additives in the food and pharmacology industries [1].

*Rubus suavissimus* S. Lee originates from the south of China and is widely planted in the Guangxi and Guizhou provinces. Due to its sweet taste, the leaves of *Rubus suavissimus* S. Lee are commonly used in Chinese sweet tea, which is locally referred to as Tiancha [2]. Its sweet taste is mainly attributed to rubusoside, a diterpene glucoside, which is 115 times sweeter than sucrose [3]. Chinese sweet tea is generally used in folk medicine for a wide range of diseases—i.e., coughs, diabetes, and atherosclerosis (high blood pressure)—as well as to promote kidney function. Moreover, different biological activities of Chinese sweet tea—i.e., antioxidant, anti-allergic, anti-inflammatory, anticancer activity, antiangiogenic, antidiabetic, and anti-obesity activities—have been reported [3,4,5,6]. These biological activities are linked to the presence of various bioactive components in Chinese sweet tea, including gallic acid, ellagic acid, rutin, kaempferol, caffeic acid, quercetin, rubusoside, and steviol monoside [3]. Recently, 14 new phenolic compounds in *Rubus suavissimus* S. Lee, including protocatechuic acid, myketin, epicatechin, vanillic acid, apigenin, catechin, ferulic acid, luteolin, 3, 3′-di-O-methylellagic acid, chlorogenic acid, 3, 3′-di-O-methylellagic acid-4′-O-β-D-glucoside, cinnamic acid, and syringate, have been identified [5].

Novel dairy products with multifunctional properties and health-promoting benefits are in high demand due to the increase in consumer health awareness. Yogurt is the most popular fermented milk, and although it has a high nutritional value and health benefits, it is a poor source of phenolic compounds. Therefore, yogurt fortified with plant materials as a natural source of phytochemicals is a new trend to increase its nutritional and functional properties. In this context, various studies have been carried out to produce yogurt supplemented with plant extracts that are rich in phytochemicals. Most of these studies have focused on the antioxidant activity of the supplemented yogurt as a direct effect of the addition of plant extracts. For instance, grape and grape callus extracts [7], moringa extract [8], cinnamon powder [9], and green and black teas [10,11,12] have been used to produce yogurt with a high antioxidant activity. Furthermore, grape seed and *Siraitia grosvenorii* fruit extracts, as a source of phytochemicals, have been used to prepare functional yogurts with high biological activities [13,14]. Nevertheless, only a few studies have reported the indirect effects of the addition of plant extracts on the angiotensin I-converting enzyme-inhibitory activity—for example, yogurt with *Mentha piperita*, *Anethum graveolens,* and *Ocimum basilicum* extracts [15], and yogurt with Raftiline HP^®^, a high-performance inulin material [16]. Additionally, the anticancer activity of yogurt has been shown to increase as an indirect effect of the addition of pineapple peel powder [17].

It is worth noting that studies on the anticancer and antihypertensive activities of yogurt supplemented with plant extracts are scarce and, to the best of our knowledge, the addition of Chinese sweet tea to milk products has not yet been reported. Accordingly, the aim of the present work was to evaluate the functional properties, including the antioxidant, anticancer, and antihypertensive activities, as well as the culture viability and sensory evaluation of yogurt fortified with Chinese sweet tea extract powder.

## 2. Materials and Methods

### 2.1. Chemicals

2,2’-azino-bis (3-ethylbenzothiazoline-6-sulfonic acid) (ABTS), gallic acid, and 1,1-diphenyl-2-picrylhydrazyl (DPPH) were purchased from Merck (Sigma Aldrich, Beijing, China). De Man Rogosa Sharpe (MRS) and M17 media were obtained from Thermo Fisher Biochemicals (Shanghai, China).

### 2.2. Preparation of the Chinese Sweet Tea Extract

Air-dried *Rubus suavissimus* S. Lee (Chinese sweet tea) leaves (local market, Nanning city, China) were extracted with boiling water (1:15 *w/v*) for 60 min, as previously described [3]. The boiled mixture was centrifuged at 5000× *g* for 15 min at room temperature and then filtered through Whatman filter paper no. 1. The final filtrate was then freeze-dried. The Chinese sweet tea extract powder was analyzed for the protein, fat, ash, and total solid contents, as previously described [18], while the carbohydrate content was calculated by the differences.

### 2.3. High-Performance Liquid Chromatography (HPLC) Analysis of Phenolic Compounds

The identification and quantification of the individual phenolic compounds were carried out using an Agilent-1260 HPLC (USA) equipped with a C18 column (Kinetex^®^ 5 µm EVO 100 × 4.6 mm, Phenomenex, Torrance, CA, USA) [19]. In brief, 5 g of Chinese sweet tea extract was dissolved in 50 mL of methanol and centrifuged at 6000× *g* for 10 min. After filtration (0.22 µm syringe filter, Minisart^®^, Sartorius Stedim Boitech, Beijing, China), 20 μL of the filtrate was injected and separated by isocratic elution of water with 0.2% phosphoric acid (*v/v*) (A; 96%), methanol (B; 2%), and acetonitrile (C; 4%) at a 0.7 mL/min flow rate. The phenolic compounds were monitored at 280 nm using a UV detector. The quantification of the phenolic compounds was calculated with external calibration through the data analysis system of the Agilent software. All the phenolic standards were provided by Merck (Sigma Aldrich, Beijing, China).

### 2.4. Yogurt Manufacture

Yogurt was produced according to the method of Abdel-Hamid et al. [14]. In brief, skimmed buffalo milk (Guangxi Buffalo Research Institute Farm, Nanning, China) was heat-treated at 90 °C for 10 min and then rapidly cooled to 43 °C. The heat-treated milk was inoculated with yogurt culture (YO-MIX 300, Danisco, China), a mix of *Lactobacillus delbrueckii ssp. bulgaricus*, and *Streptococcus thermophilus,* according to the manual provided by the manufacturer. The cultured milk was divided into four equal parts: one part was used as a control treatment and the other three parts were incorporated with the Chinese sweet tea extract powder at concentrations of 0.25%, 0.5%, and 1% (*w/w*). Each milk portion was poured into plastic containers (120 mL) and incubated at 42 °C until the pH was reduced to 4.6. The yogurt treatments were kept at 4 ± 1 °C.

### 2.5. Chemical Composition

The carbohydrate, protein, and total solid contents of the cultured milk combined with the Chinese sweet tea extract powder were measured by a MilkoScan analyzer (F120, FOSS, Hillerød, Denmark). The pH values of the yogurt samples were monitored using a digital pH meter (Methrohm AG, Herisau, Switzerland).

### 2.6. Bacterial Counts

Bacterial counts of the yogurt samples were performed after 24 h of storage at 4 °C using the pour plate method [14]. *S. thermophilus* was grown on M17 agar, and the plates were aerobically incubated at 37 °C for 24 h. *L. bulgaricus* was enumerated on MRS agar (pH 5.4), and the plates were anaerobically incubated at 37 °C for 24 h. The results are presented as log colony-forming units per gram yogurt (CFU/g).

### 2.7. Yogurt Supernatant

The yogurt supernatant was separated by centrifugation at 22,000× *g* for 30 min at 4 °C, followed by filtration using a 0.45 µm syringe filter (Minisart^®^, Sartorius Stedim Boitech, Beijing, China). The filtrate of the yogurt samples was used to evaluate the total phenolic content and the biological activities—i.e., the antioxidant, anticancer, and antihypertensive activities.

### 2.8. Total Phenolic Content (TPC)

The TPC of the Chinese sweet tea extract powder and the yogurt samples was measured using the Folin–Ciocalteu assay [8]. Thirty microliters of the yogurt supernatant or the Chinese sweet tea extract powder (1 mg/mL in water) were pipetted into a 96-well plate, and then distilled water (120 µL) and Folin–Ciocalteu’s phenol reagent (30 µL) were added, respectively, to each well, followed by 30 µL of sodium carbonate (1N). The plates were incubated in the dark for 30 min at room temperature, and then the absorbance was read at 750 nm using a microplate reader (EPOCH, BioTek, Winooski, VT, USA). A standard curve was constructed using different concentrations of gallic acid. The results are expressed as the µg gallic acid equivalents (GAE) per milliliter.

### 2.9. Antioxidant Activity

ABTS and DPPH radical scavenging assays were used to evaluate the antioxidant activity of the yogurt samples according to Abdel-Hamid et al. [14]. In short, 50 µL of the yogurt supernatant was pipetted into a 96-well plate followed by ABTS^+^ solution (200 µL). The plate was then incubated in the dark for 30 min, and the absorbance was monitored at 405 nm using a microplate reader. 

For the DPPH assay, DPPH reagent (0.2 mM) was freshly prepared and added to the yogurt supernatant (1:1 *v/v*). After a 30 min reaction in the dark at 37 °C, the absorbance was measured at 517 nm.

### 2.10. Anticancer Activity

The anticancer activity of the yogurt samples was assessed against the Caco-2 carcinoma cell line (HTB-37; American Type Culture Collection, Manassas, VA, USA), and the cells were propagated according to Abdel-Hamid et al. [20]. Caco-2 cells were plated into 96-well plates (3000 cells/well) and incubated overnight at 37 °C under 5% CO_2_. The cells were then treated with 25 µL of the yogurt supernatant and grown again for 24 h. The viability of the treated cells was evaluated by the WST assay. The antiproliferative activity was calculated using the following equation:

Antiproliferative activity (%) = [1 − (A − B)/(C − B)] × 100,(1)

where A is the absorbance of the cells in the presence of the yogurt supernatant, B is the background absorbance (non-cell control), and C is the absorbance of the control (cells with sterilized water instead of the yogurt supernatant).

### 2.11. Antihypertensive Activity

The antihypertensive activity of the Chinese sweet tea extract and the yogurt samples was investigated by measuring the angiotensin-converting enzyme (ACE) inhibitory activity. The spectrophotometric method was employed to evaluate the ACE inhibition by the Chinese sweet tea extract and the yogurt samples using the ACE Kit-WST (Dojindo laboratories, Shanghai, China) [20].

### 2.12. Sensory Evaluation

Samples of the yogurt with and without Chinese sweet tea extract were characterized organoleptically after 24 h of cold storage at 4 ± 1 °C by seven trained panelists (researchers at Guangxi University, Nanning, China) with an interest and experience in the sensory evaluation of fermented milk. A 9-point scale was used to evaluate the yogurt samples in terms of their appearance, aroma, texture, and overall acceptability, as described by Romeih et al. [21].

### 2.13. Statistical Analysis

Three independent experiments were performed in this study, and the results are presented as the mean ± standard deviation (SD). A one-way analysis of variance (ANOVA) was carried out with Statistix 8.1 (Analytical Software, Tallahassee, FL, USA) using Tukey’s test for pairwise comparison. The correlation between variables was evaluated by the Pearson correlation test.

## 3. Results and Discussion

### 3.1. Impact of the Chinese Sweet Tea Extract on the Chemical Composition of the Yogurt Samples

The chemical characterization of the Chinese sweet tea extract powder is presented in Table 1. The Chinese sweet tea extract powder contained 94.22 ± 1% total solids, 6.4 ± 0.25% protein, and 80.18 ± 1.2% total carbohydrates. The chemical composition of the heat-treated milk with the Chinese sweet tea extract before fermentation was measured using the MilkoScan, and the results are presented in Table 2. The addition of the Chinese sweet tea extract had no significant effect (*p* > 0.05) on the protein content of the yogurt samples, whereas the total solid and total carbohydrate contents were significantly increased (*p* ˂ 0.05) compared with the control yogurt sample. These results might be attributed to the high contents of carbohydrates and total solids in the Chinese sweet tea extract powder. It has been reported that the crude water extract of Chinese sweet tea contains 11% polysaccharides [2]. A similar trend was observed for the total carbohydrate and total solid contents in yogurt supplemented with *Siraitia grosvenorii* fruit, moringa, and *Gnaphalium affine* extracts [8,14,22].

Regarding the TPC, the Chinese sweet tea extract powder contained 21.54 ± 0.55 mg GAE/g (Table 1). The yogurt with the Chinese sweet tea extract had a significantly higher TPC content (*p* ˂ 0.05) compared to the control yogurt sample, which gradually increased with an increase in the amount of Chinese sweet tea extract (Table 2). The highest TPC content (130.58 µg GAE/mL) was measured in the yogurt sample containing 1% Chinese sweet tea extract. These results are in agreement with those of Amirdivani and Baba [12], Gao et al. [22], Karaaslan et al. [7], and Zhang et al. [8], who found significantly increasing TPC contents in yogurt fortified with green tea, moringa, *Gnaphalium affine,* grape, and callus extracts. It should be noted that the TPC contents reported in these studies were lower than those detected in our study (130.58 µg GAE/mL).

### 3.2. Identification and Quantification of Phenolic Compounds in the Chinese Sweet Tea Extract

The phenolic compounds were determined using the HPLC assay, and the results are presented in Table 3. A total of 19 phenolic compounds were identified and quantified in the Chinese sweet tea extract powder, with concentrations ranging from 0.08 to 2.77 mg/g. Benzoic acid, quercetin, rutin, syringic acid, ellagic acid, and gallic acid were the most abundant phenolic compounds detected in the Chinese sweet tea extract. Indeed, Koh et al. [3] reported ellagic acid, rutin, and gallic acid as the major phenolic compounds of Chinese sweet tea, and they used the rubusoside content to evaluate the quality of the Chinese sweet tea. It has been reported that the concentrations of ellagic acid, rutin, and gallic acid in 14 Chinese sweet tea samples collected in different seasons ranged from 0.46% to 92%, 0.08% to 0.15%, and 0.1% to 0.16%, respectively [2]. More recently, Liu et al. [5] reported 14 new phenolic compounds in Chinese sweet tea leaves. In our study, seven new phenolic compounds were identified for the first time in Chinese sweet tea namely, *p*-coumaric acid, benzoic acid, *o*-coumaric acid, resveratrol, neringein, rosmarinic, and myricetin. These findings suggest that the phenolic compound content varies according to the region and season of growth [2].

### 3.3. pH and Bacterial Count

The addition of the Chinese sweet tea extract increased the fermentation time from 5 h in the control yogurt sample to 6 h in the yogurt sample containing the 1% Chinese sweet extract. This was probably due to the antibacterial activity of the Chinese sweet tea components toward lactic acid bacteria, along with its acknowledged inhibition of pathogenic bacteria [13,23]. Additionally, Chinese sweet tea contains a high amount of benzoic acid, which is known to be an antimicrobial agent (Table 3). Likewise, the fermentation time was not affected by the addition of green and black teas [10]. In contrast, it has been reported that the addition of moringa, *Mentha piperita*, *Anethum graveolens*, or *Ocimum basilicum* extracts to yogurt shortens the fermentation time due to the enhancement of the growth of yogurt cultures [8,15].

The pH values of the yogurt samples after 24 h of cold storage are presented in Table 4. The addition of the Chinese sweet tea extract had no significant effect (*p* > 0.05) on the pH values. In agreement with this finding, the use of different types of spices (i.e., cardamom, cinnamon, or nutmeg), moringa, and cinnamon and licorice herbals in the preparation of yogurt had no significant effect on the pH values compared with the relevant control yogurt [8,23,24].

The viability of the yogurt cultures of *S. thermophilus* and *L. bulgaricus* is presented in Table 4 as the log CFU/g yogurt. The viable cell counts of *L. bulgaricus* and *S. thermophilus* in the yogurt samples were 8.6–8.9 and 9.1–9.3 log CFU/g, respectively. The viable counts of *L. bulgaricus* and *S. thermophilus* in the yogurt samples are higher than the recommended dose to promote health benefits (>6 log CFU/g) [25]. Yogurt samples fortified with Chinese sweet tea had no significant influence (*p >* 0.05) on the viability of *L. bulgaricus* and *S. thermophilus.* A similar trend was reported for the addition of strawberries and green or black teas before yogurt fermentation [10,26]. Behrad et al. [23] reported that yogurt mixed with cinnamon and licorice herbals had lower counts of *L. bulgaricus* and *S. thermophilus* compared to the control plain yogurt. In contrast, the addition of Japanese and Malaysian green teas or moringa extract significantly improved the viability of *L. bulgaricus* and *S. thermophilus* in yogurt [8,12]. These findings demonstrate that the effects of the addition of extracts on the fermentation time, pH values, and culture viability of yogurt depend on the plant type and the phytochemical concentrations.

### 3.4. Antioxidant Activity

The antioxidant activities of the yogurt samples were measured by ABTS and DPPH assays, and the results are presented in Table 5. The antioxidant activity of the yogurt samples ranged between 14.2% and 74.83% for the DPPH assay and between 32.01% and 92.43% for the ABTS assay. The addition of Chinese sweet tea extract significantly increased (*p* ˂ 0.05) the antioxidant activity as the amount of extract added increased. The yogurt sample containing 1% Chinese sweet tea extract showed the highest ABTS (92.43%) and DPPH (74.83%) values. A positive correlation was observed between the TPC and the antioxidant activity for the DPPH (*r* = 0.998) and ABTS (*r* = 0.993) assays. Shori et al. [27] reported a similar trend between the TPC and antioxidant activity of phytomix-3+ mangosteen (a mixture of *Lycium barbarum*, *Momordica grosvenori,* and *Psidium guajava* leaves) yogurt. The increase in the antioxidant activities of yogurt fortified with Chinese sweet tea extract could be due to the higher concentrations of phytochemicals (i.e., phenols, flavonoids, and rubusoside) in Chinese sweet tea [2]. Our results are in agreement with those of Amirdivani and Baba [12] and Najgebauer-Lejko et al. [11], who found significantly higher antioxidant activities in yogurt supplemented with Japanese green tea, Malaysian green tea, green tea, or Pu-erh tea compared to the control yogurt. Furthermore, the addition of moringa, *Mentha piperita*, *Anethum graveolens*, *Ocimum basilicum*, spice oleoresins (i.e., cardamom, cinnamon, and nutmeg), grape seed, and cinnamon and licorice herbal extracts significantly increases the antioxidant activity of yogurt [8,9,13,15,23,24]. These authors concluded that the increase in antioxidant activities is a result of the concentrations of phenolic compounds in the plant extracts.

It is worth noting that the antioxidant activities of the yogurt samples containing Chinese sweet tea extract measured by the DPPH (29.8–74.8%) and ABTS (49.4–92.4%) assays were higher than the antioxidant activities of the yogurt produced by the above-mentioned studies. This may be attributed to the differences in the phytochemical types and their concentrations.

### 3.5. Anticancer Activity

The anticancer activity of the yogurt samples was examined by measuring the ability of the yogurt supernatant to inhibit the proliferation of the Caco-2 cell line, and the results are presented in Table 6. The Chinese sweet tea extract exhibited a 89.06% ± 3.7% anticancer activity at a concentration of 0.4 mg/mL (Table 1). The control yogurt sample also showed anticancer activity, which is most probably attributed to the presence of bioactive peptides. A similar finding was reported by Sah et al. [17]. Although the addition of 0.25% Chinese sweet tea extract had no significant impact (*p* > 0.05) on the anticancer activity levels compared to the control yogurt sample, yogurt samples containing 0.5% and 1% Chinese sweet tea extract exhibited significantly higher (*p* ˂ 0.05) anticancer activity than that of the control yogurt sample (Table 5). Moreover, the supernatant of the yogurt sample containing 1% Chinese sweet tea extract showed the highest anticancer activity and inhibited the growth of the Caco-2 cell by 67.46%. It is worth noting that the anticancer activity was positively correlated *(r* = 0.961) with the TPC contents of the yogurts. This finding is probably attributed to the phytochemical content of the Chinese sweet tea extract, including phenolic compounds and rubusoside [2,3]. George Thompson et al. [28] reported that rubusoside, the main component in Chinese sweet tea (5% in dry leaves), inhibits the glucose (GLUT1) and fructose (GLUT5) transporters associated with cancer and diabetes. In addition, quercetin, rutin, and ellagic acid, which are among the major phenolic compounds in Chinese sweet tea, show anticancer activity against colon carcinoma cells, as described by Hashemzaei et al. [29] and Papoutsi et al. [30]. In accordance with our results, Sah et al. [17] reported the potential anticancer activity of probiotic yogurt supplemented with pineapple peel powder against HT29 colon cancer cells compared to the control. These authors attributed this finding to the enhanced extent of proteolysis and, consequently, the resultant bioactive peptides released by the addition of pineapple peel powder to the yogurt.

### 3.6. Antihypertensive Activity

The antihypertensive activity was evaluated using the ACE inhibition method, as ACE is a key factor in the conversion of angiotensin I to angiotensin II, which raises blood pressure [31]. Chinese sweet tea extract exhibited a 86.85% ± 2.1% ACE inhibitory activity at 0.4 mg/mL (Table 1). The ACE inhibitory activity of the yogurt samples is shown in Table 6. The yogurt samples without the addition of Chinese sweet tea also showed an ACE inhibitory activity, which may due to the presence of bioactive peptides with ACE inhibitory activities already known to be present in yogurt products. In this context, Abdel-Hamid et al. [14] and Amirdivani and Baba [15] also reported ACE inhibitory activity for their control yogurts. As can be seen in Table 6, the addition of Chinese sweet tea extract significantly enhanced (*p* ˂ 0.05) the ACE inhibitory activity of the yogurt samples. The ACE inhibitory activity was increased by increasing the amount of Chinese sweet tea extract added, where the yogurt sample containing 1% Chinese sweet tea extract showed the highest value (82.03%). This finding could be due to the bioactive compounds in the Chinese sweet tea extract. It should be noted that quercetin, rutin, and gallic acid, the major phenolic compounds in the Chinese sweet tea extract, exhibit antihypertensive activity both in vitro and in vivo, as reported by Balasuriya and Rupasinghe [32], Kang et al. [33], and Shaw et al. [34]. In addition, the ACE inhibitory activity of the yogurt samples was positively correlated with the TPC (*r* = 0.994). It has been reported that Chinese sweet tea exhibits antihypertensive activity, which can be attributed to its phytochemical content [35]. These results are in accordance with those reported by Liu and Finley [36], who concluded that phytochemicals reduce blood pressure. Furthermore, *Chrysophyllum cainito* fruit extract, rich in polyphenols, shows ACE inhibitory activity by chelating Zn^2+^ ions (enzyme cofactor) [31]. Amirdivani and Baba [15] reported that yogurt fortified with herbal water extracts (i.e., *Mentha piperita, Anethum graveolens,* and *Ocimum basilicum*) exhibited a higher ACE inhibitory activity compared to the control yogurt. Moreover, the addition of Raftiline HP^®^ has been shown to significantly improve the ACE inhibitory activity compared to plain yogurt [16]. These authors attributed the increase in ACE inhibitory activity after the addition Raftiline HP^®^ or herbal water extracts to the higher degree of proteolysis of the fortified yogurts, resulting in bioactive peptides with ACE inhibitory activity. However, Amirdivani and Baba [15] reported that the water extract of *Mentha piperita, Anethum graveolens,* or *Ocimum basilicum* itself had no ACE inhibitory activity. Interestingly, the obtained ACE inhibitory activity values of yogurts prepared with Chinese sweet tea extracts were higher than those reported by Amirdivani and Baba [15] and Ramchandran and Shah [16].

It should be noted that the Chinese sweet tea extract had no effect on either the viability of the yogurt culture (Table 4) or the degree of hydrolysis (data not shown). However, the Chinese sweet tea extract exhibited potential anticancer and antihypertensive activities (Table 1). Accordingly, the biological activities (i.e., antioxidant, antihypertensive, and anticancer activities) of the yogurt samples containing Chinese sweet tea extract are most probably attributed to the phytochemicals present in Chinese sweet tea.

### 3.7. Sensory Evaluation

Sensory characteristics are very important for assessing the consumer acceptability of yogurt products and to confirm that the additives have no negative impacts on the organoleptic parameters of said yogurt products. The sensory evaluation of the yogurt samples fortified with Chinese sweet tea extract is shown in Table 7. The yogurt samples containing Chinese sweet tea extract received almost similar appearance scores as the control yogurt sample (*p* > 0.05). The addition of the Chinese sweet tea extract significantly increased (*p* ˂ 0.05) the texture score. Furthermore, the addition of Chinese sweet tea significantly enhanced (*p* < 0.05) the aroma of the yogurt samples compared to the control yogurt sample, which could be due to the sweet taste of rubusoside. In particular, the yogurt sample containing 1% Chinese sweet tea extract received the lowest perceived aroma score (*p* < 0.05) compared to the samples containing 0.25% and 0.5% Chinese sweet tea extract and the control yogurt sample. This finding may be attributed to the bitter aftertaste of rubusoside and some phenolic compounds [2,3]. Similarly, the overall acceptability score of the yogurt samples containing 0.25% and 0.5% Chinese sweet extract was significantly higher (*p* ˂ 0.05) than the control yogurt, while the yogurt sample containing 1% Chinese sweet tea extract received the lowest overall acceptability score. In this context, yogurt prepared with moringa extract at different levels (i.e., 0.05%, 0.1%, and 0.2%) had a significantly lower sensory evaluation compared to the control yogurt [8]. The authors attributed these results to the better taste of moringa. The above results demonstrate that the yogurt samples with 0.5% Chinese sweet tea extract had the best sensory evaluation. 

## 4. Conclusions

This study aimed to produce a multifunctional yogurt rich in phytochemicals using Chinese sweet tea extract. The addition of the Chinese sweet tea extract did not influence the viability of the yogurt culture but significantly enhanced the biological activities of the yogurt samples, including their antioxidant, anticancer, and antihypertensive activities. The addition of Chinese sweet tea at concentrations of 0.25% and 0.5% significantly improved the aroma of the yogurt samples compared to the control yogurt sample. Although the yogurt sample containing 1% Chinese sweet tea extract exhibited the highest antioxidant, anticancer, and antihypertensive activities, it received the lowest aroma score due to the bitter aftertaste of rubusoside. Overall, the yogurt sample containing 0.5% Chinese sweet tea extract had significantly higher biological activities and sensory evaluation compared to the control yogurt sample. Therefore, 0.5% Chinese sweet tea extract appears to be an efficient option and a promising ingredient for producing a multifunctional yogurt rich in phytochemicals with health-promoting properties.

## Figures and Tables

**Table 1:** Characterization of the Chinese sweet tea extract.

Characteristic	Chinese Sweet Tea Extract
Total solid (%)	94.22 ± 1.0
Protein (%)	6.40 ± 0.25
Fat (%)	0.42 ± 0.1
Ash (%)	7.21 ± 0.1
Carbohydrates (%)	80.18 ± 1.2
Total phenolic content *	21.54 ± 0.5
ACE-I activity (%)	86.85 ± 2.1
Anticancer activity (%)	89.06 ± 3.7

Values are the mean of three replicates ± standard deviation. * Total phenolic content presented as mg of gallic acid equivalents (GAE) per gram of Chinese sweet tea extract powder. ACE-I, angiotensin-converting enzyme-inhibition.

**Table 2:** Chemical composition of heat-treated milk fortified with Chinese sweet tea extract.

Chinese Sweet Tea Extract Concentration	Protein (%)	Carbohydrates (%)	Total Solids (%)	Total Phenolic Content *
0% (Control)	5.10 ± 0.08 ^A^	6.17 ± 0.01 ^D^	12.31 ± 0.02 ^D^	11.64 ± 1.2 ^D^
0.25%	5.15 ± 0.02 ^A^	6.26 ± 0.02 ^C^	12.56 ± 0.02 ^C^	41.60 ± 2.0 ^C^
0.5%	5.16 ± 0.02 ^A^	6.35 ± 0.01 ^B^	12.84 ± 0.04 ^B^	70.93 ± 2.2 ^B^
1%	5.18 ± 0.02 ^A^	6.59 ± 0.01 ^A^	13.48 ± 0.02 ^A^	130.58 ± 2.2 ^A^

Results are the mean of three experiments ± standard deviation. Values in the same column with different superscript letters are significantly different (*p* ˂ 0.05). * Total phenolic content presented as µg gallic acid equivalents (GAE) per milliliter of yogurt supernatant.

**Table 3:** Identified phenolic compounds in the Chinese sweet tea extract.

Phenolics	Concentration (mg/g)
Gallic acid	1.26 ± 0.006
Catchin	0.08 ± 0.001
Chlorgenic	0.66 ± 0.003
Vanillic acid	0.96 ± 0.001
Caffeic acid	0.47 ± 0.005
Syringic acid	1.53 ± 0.006
*p*-Coumaric acid	1.08 ± 0.006
Benzoic acid	2.77 ± 0.007
Ferulic acid	0.11 ± 0.001
Rutin	1.63 ± 0.004
Ellagic	1.44 ± 0.007
*o*-Coumaric acid	0.74 ± 0.004
Resveratrol	0.52 ± 0.003
Cinnamic acid	0.11 ± 0.001
Quercetin	1.98 ± 0.005
Neringein	0.59 ± 0.004
Rosemarinic	0.55 ± 0.005
Myricetin	0.46 ± 0.005
Kampherol	0.38 ± 0.004

Values are the mean of three replicates ± standard deviation.

**Table 4:** Viability of the yogurt cultures (log CFU/g) and pH values of the yogurt fortified with Chinese sweet tea extract.

Chinese Sweet Tea Extract Concentration	pH	*L. bulgaricus* (log CFU/g)	*S. thermophilus* (log CFU/g)
0% (Control)	4.52 ± 0.02 ^A^	8.65 ± 0.1 ^A^	9.20 ± 0.04 ^A^
0.25%	4.53 ± 0.02 ^A^	8.83 ± 0.03 ^A^	9.22 ± 0.03 ^A^
0.5%	4.54 ± 0.03 ^A^	8.90 ± 0.04 ^A^	9.25 ± 0.1 ^A^
1%	4.54 ± 0.02 ^A^	8.86 ± 0.5 ^A^	9.18 ± 0.02 ^A^

Results are the mean of three experiments ± standard deviation. Values in the same column with different superscript letters are significantly different (*p* ˂ 0.05).

**Table 5:** Antioxidant activity of the yogurt fortified with Chinese sweet tea extract.

Chinese Sweet Tea Extract Concentration	Antioxidant Activity (%)	
	ABTS	DPPH
0% (Control)	32.01 ± 1.0 ^D^	14.20 ± 0.4 ^D^
0.25%	49.40 ± 0.9 ^C^	29.85 ± 1.4 ^C^
0.5%	68.76 ± 2.2 ^B^	47.29 ± 2.2 ^B^
1%	92.43 ± 0.3 ^A^	74.83 ± 0.9 ^A^

Results are the mean of three experiments ± standard deviation. Values in the same column with different superscript letters are significantly different (*p* ˂ 0.05).

**Table 6:** Antiproliferative and ACE-I activities of the yogurt fortified with Chinese sweet tea extract.

Sweet Tea Extract Concentration	ACE-I (%)	Antiproliferative Activity (%)
0% (Control)	44.20 ± 0.7 ^D^	18.58 ± 1.4 ^C^
0.25%	50.88 ± 1.1 ^C^	26.27 ± 4.5 ^C^
0.5%	64.72 ± 1.6 ^B^	52.41 ± 6.1 ^B^
1%	82.03 ± 1.9 ^A^	67.46 ± 1.9 ^A^

Results are the mean of three experiments ± standard deviation. Values in the same column with different superscript letters are significantly different (*p* ˂ 0.05).

**Table 7:** Sensory evaluation of yogurt fortified with Chinese sweet tea extract.

Chinese Sweet Tea Extract Concentration	Appearance	Aroma	Texture	Overall Acceptability
0% (Control)	7.7 ± 0.5 ^A^	6.9 ± 0.4 ^C^	6.0 ± 0.8 ^C^	6.7 ± 0.8 ^C^
0.25%	8.0 ± 0.7 ^A^	7.7 ± 0.5 ^B^	6.6 ± 0.5 ^C^	7.6 ± 0.5 ^B^
0.5%	8.1 ± 0.8 ^A^	8.6 ± 0.5 ^A^	7.6 ± 0.5 ^B^	8.7 ± 0.5 ^A^
1%	8.3 ± 0.8 ^A^	6.0 ± 0.6 ^D^	8.7 ± 0.5 ^A^	5.7 ± 0.5 ^D^

Results are the mean of three experiments ± standard deviation. Values in the same column with different superscript letters are significantly different (*p* ˂ 0.05).

## References

[1] Khoshnoudi-Nia S., Sharif N., Jafari S.M. (2020). Loading of phenolic compounds into electrospun nanofibers and electrosprayed nanoparticles. Trends Food Sci. Technol..

[2] Chou G., Xu S.-J., Liu D., Koh G.Y., Zhang J., Liu Z. (2009). Quantitative and fingerprint analyses of Chinese sweet tea plant (*Rubus suavissimus* S. Lee). J. Agric. Food Chem..

[3] Koh G.Y., Chou G., Liu Z. (2009). Purification of a water extract of Chinese sweet tea plant (Rubus suavissimus S. lee) by alcohol precipitation. J. Agric. Food Chem..

[4] Ezure T., Amano S. (2011). Rubus suavissimus S. Lee extract increases early adipogenesis in 3T3-L1 preadipocytes. J. Nat. Med..

[5] Liu M., Li X., Liu Q., Xie S., Chen M., Wang L., Feng Y., Chen X. (2020). Comprehensive profiling of α-glucosidase inhibitors from the leaves of Rubus suavissimus using an off-line hyphenation of HSCCC, ultrafiltration HPLC-UV-MS and prep-HPLC. J. Food Compos. Anal..

[6] Zhang H., Qi R., Zeng Y., Tsao R., Mine Y. (2020). Chinese sweet leaf tea (Rubus suavissimus) mitigates LPS-induced low-grade chronic inflammation and reduces the risk of metabolic disorders in a C57BL/6J mouse model. J. Agric. Food Chem..

[7] Karaaslan M., Ozden M., Vardin H., Turkoglu H. (2011). Phenolic fortification of yogurt using grape and callus extracts. LWT—Food Sci. Technol..

[8] Zhang T., Jeong C.H., Cheng W.N., Bae H., Seo H.G., Petriello M.C., Han S.G. (2019). Moringa extract enhances the fermentative, textural, and bioactive properties of yogurt. LWT—Food Sci. Technol..

[9] Helal A., Tagliazucchi D. (2018). Impact of in-vitro gastro-pancreatic digestion on polyphenols and cinnamaldehyde bioaccessibility and antioxidant activity in stirred cinnamon-fortified yogurt. LWT—Food Sci. Technol..

[10] Jaziri I., Ben Slama M., Mhadhbi H., Urdaci M.C., Hamdi M. (2009). Effect of green and black teas (*Camellia sinensis* L.) on the characteristic microflora of yogurt during fermentation and refrigerated storage. Food Chem..

[11] Najgebauer-Lejko D., Sady M., Grega T., Walczycka M. (2011). The impact of tea supplementation on microflora, pH and antioxidant capacity of yoghurt. Int. Dairy J..

[12] Amirdivani S., Baba A.S.H. (2015). Green tea yogurt: Major phenolic compounds and microbial growth. J. Food Sci. Technol..

[13] Chouchouli V., Kalogeropoulos N., Konteles S.J., Karvela E., Makris D.P., Karathanos V.T. (2013). Fortification of yoghurts with grape (*Vitis vinifera*) seed extracts. LWT—Food Sci. Technol..

[14] Abdel-Hamid M., Romeih E., Huang Z., Enomoto T., Huang L., Li L. (2020). Bioactive properties of probiotic set-yogurt supplemented with Siraitia grosvenorii fruit extract. Food Chem..

[15] Amirdivani S., Baba A.S. (2011). Changes in yogurt fermentation characteristics, and antioxidant potential and in vitro inhibition of angiotensin-1 converting enzyme upon the inclusion of peppermint, dill and basil. LWT—Food Sci. Technol..

[16] Ramchandran L., Shah N.P. (2008). Growth, proteolytic, and ACE-I activities of Lactobacillus delbrueckii ssp. bulgaricus and Streptococcus thermophilus and rheological properties of low-fat yogurt as influenced by the addition of Raftiline HP^®^. J. Food Sci..

[17] Sah B.N.P., Vasiljevic T., McKechnie S., Donkor O.N. (2016). Antibacterial and antiproliferative peptides in synbiotic yogurt-Release and stability during refrigerated storage. J. Dairy Sci..

[18] (2000). AOAC Official Methods of Analysis of AOAC International.

[19] Türköz Acar E., Celep M.E., Charehsaz M., Akyüz G.S., Yeşilada E. (2018). Development and validation of a high-performance liquid chromatography–diode-array detection method for the determination of eight phenolic constituents in extracts of different wine species. Turk. J. Pharm. Sci..

[20] Abdel-Hamid M., Romeih E., Gamba R.R., Nagai E., Suzuki T., Koyanagi T., Enomoto T. (2019). The biological activity of fermented milk produced by Lactobacillus casei ATCC 393 during cold storage. Int. Dairy J..

[21] Romeih E.A., Abdel-Hamid M., Awad A.A. (2014). The addition of buttermilk powder and transglutaminase improves textural and organoleptic properties of fat-free buffalo yogurt. Dairy Sci. Technol..

[22] Gao H.-X., Yu Z.-L., He Q., Tang S.-H., Zeng W.-C. (2018). A potentially functional yogurt co-fermentation with Gnaphalium affine. LWT—Food Sci. Technol..

[23] Behrad S., Yusof M.Y., Goh K.L., Baba A.S. (2009). Manipulation of probiotics fermentation of yogurt by cinnamon and licorice: Effects on yogurt formation and inhibition of Helicobacter pylori growth in vitro. World Acad. Sci. Eng. Technol..

[24] Illupapalayam V.V., Smith S.C., Gamlath S. (2014). Consumer acceptability and antioxidant potential of probiotic-yogurt with spices. LWT—Food Sci. Technol..

[25] Tripathi M.K., Giri S.K. (2014). Probiotic functional foods: Survival of probiotics during processing and storage. J. Funct. Foods.

[26] Oliveira A., Alexandre E.M.C., Coelho M., Lopes C., Almeida D.P.F., Pintado M. (2015). Incorporation of strawberries preparation in yoghurt: Impact on phytochemicals and milk proteins. Food Chem..

[27] Shori A.B., Rashid F., Baba A.S. (2018). Effect of the addition of phytomix-3+ mangosteen on antioxidant activity, viability of lactic acid bacteria, type 2 diabetes key-enzymes, and sensory evaluation of yogurt. LWT—Food Sci. Technol..

[28] George Thompson A.M., Iancu C.V., Nguyen T.T.H., Kim D., Choe J. (2015). Inhibition of human GLUT1 and GLUT5 by plant carbohydrate products; insights into transport specificity. Sci. Rep..

[29] Hashemzaei M., Far A.D., Yari A., Heravi R.E., Tabrizian K., Taghdisi S.M., Sadegh S.E., Tsarouhas K., Kouretas D., Tzanakakis G. (2017). Anticancer and apoptosis-inducing effects of quercetin in vitro and in vivo. Oncol. Rep..

[30] Papoutsi Z., Kassi E., Chinou I., Halabalaki M., Skaltsounis L.A., Moutsatsou P. (2008). Walnut extract (*Juglans regia* L.) and its component ellagic acid exhibit anti-inflammatory activity in human aorta endothelial cells and osteoblastic activity in the cell line KS483. Br. J. Nutr..

[31] Mao L.M., Qi X.W., Hao J.H., Liu H.F., Xu Q.H., Bu P.L. (2015). In vitro, ex vivo and in vivo anti-hypertensive activity of *Chrysophyllum cainito* L.. Extract. Int. J. Clin. Exp. Med..

[32] Balasuriya N., Rupasinghe H.P.V. (2012). Antihypertensive properties of flavonoid-rich apple peel extract. Food Chem..

[33] Kang N., Lee J., Lee W., Ko J., Kim E., Kim J., Heu M., Hoon G., Jeon Y. (2015). Gallic acid isolated from Spirogyra sp. improves cardiovascular disease through a vasorelaxant and antihypertensive effect. Environ. Toxicol. Pharmacol..

[34] Shaw H., Wu J., Wang M. (2017). Antihypertensive effects of Ocimum gratissimum extract: Angiotensin-converting enzyme inhibitor in vitro and in vivo investigation. J. Funct. Foods.

[35] Zhu X.H., Xia M., Yang C.X. (2014). Optimizing the Extraction Process of Rubusoside from the Rubus Suavissimus: A Cellulase Pretreatment Approach. Appl. Mech. Mater..

[36] Liu R., Finley J. (2005). Potential cell culture models for antioxidant research. J. Agric. Food Chem..

